# Bis(2,4,6-triamino-1,3,5-triazin-1-ium) tris­(pyridine-2,6-dicarboxyl­ato)­zirconate(IV) tetra­hydrate

**DOI:** 10.1107/S1600536808029887

**Published:** 2008-09-20

**Authors:** Shirin Daneshvar, Hossein Aghabozorg, Faranak Manteghi

**Affiliations:** a Department of Chemistry, Islamic Azad University, Ardabil Branch, Ardabil, Iran; b Faculty of Chemistry, Tarbiat Moallem University, Tehran, Iran; c Faculty of Chemistry, Iran University of Science and Technology, Tehran, Iran

## Abstract

The title compound, (C_3_H_7_N_6_)_2_[Zr(C_7_H_3_NO_4_)_3_]·4H_2_O or (tataH)_2_[Zr(pydc)_3_]·4H_2_O (tata is 2,4,6-triamino-1,3,5-triazine and pydcH_2_ is pyridine-2,6-dicarboxylic acid), was obtained by reaction between pydcH_2_, tata and zirconyl chloride octa­hydrate in aqueous solution. In the structure, the Zr^IV ^atom is nine-coordinated by three (pydc)^2−^ groups, resulting in an anionic complex which is balanced by two (tataH)^+^ cations. One of the NH_2_ groups shows positional disorder, with site occupation factors of 0.60 and 0.40. There are four uncoordinated water mol­ecules  (one of which is disordered with occupation factors of 0.70 and 0.30) in the crystal structure. Several inter­molecular inter­actions, including O—H⋯O, O—H⋯N, N—H⋯O, N—H⋯N, C—H⋯O and C—H⋯N hydrogen bonds, a C—O⋯π inter­action [O⋯*Cg* 3.89, C⋯*Cg* 4.068 (3) Å; C—O⋯*Cg* 89° where *Cg* is the centroid of the triamine ring], and π–π stacking [with centroid–centroid distances of 3.694 (2) and 3.802 (2) Å] are also present.

## Related literature

For related literature, see: Aghabozorg *et al.* (2005 ▶, 2008 ▶); Harben *et al.* (2004 ▶); Soleimannejad *et al.* (2007 ▶).
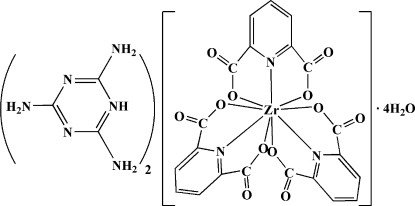


## Experimental

### 

#### Crystal data

(C_3_H_7_N_6_)_2_[Zr(C_7_H_3_NO_4_)_3_]·4H_2_O
*M* *_r_* = 912.89
Triclinic, 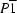

*a* = 9.3749 (16) Å
*b* = 12.308 (3) Å
*c* = 16.934 (4) Å
α = 97.926 (19)°
β = 106.050 (12)°
γ = 107.839 (11)°
*V* = 1733.8 (7) Å^3^
*Z* = 2
Mo *K*α radiation
μ = 0.42 mm^−1^
*T* = 120 (2) K
0.40 × 0.20 × 0.15 mm

#### Data collection

Bruker SMART 1000 CCD area-detector diffractometer
Absorption correction: multi-scan (*SADABS*; Sheldrick, 1996 ▶) *T*_min_ = 0.851, *T*_max_ = 0.940
17993 measured reflections
8355 independent reflections
6766 reflections with *I* > 2σ(*I*)
*R*_int_ = 0.027

#### Refinement

*R*[*F* ^2^ > 2σ(*F* ^2^)] = 0.041
*wR*(*F* ^2^) = 0.104
*S* = 1.00
8355 reflections
545 parameters
H-atom parameters constrained
Δρ_max_ = 1.00 e Å^−3^
Δρ_min_ = −0.77 e Å^−3^

### 

Data collection: *SMART* (Bruker, 2007 ▶); cell refinement: *SAINT* (Bruker, 2007 ▶); data reduction: *SAINT*; program(s) used to solve structure: *SHELXTL* (Sheldrick, 2008 ▶); program(s) used to refine structure: *SHELXTL*; molecular graphics: *SHELXTL*; software used to prepare material for publication: *SHELXTL*.

## Supplementary Material

Crystal structure: contains datablocks I, global. DOI: 10.1107/S1600536808029887/om2259sup1.cif

Structure factors: contains datablocks I. DOI: 10.1107/S1600536808029887/om2259Isup2.hkl

Additional supplementary materials:  crystallographic information; 3D view; checkCIF report

## Figures and Tables

**Table 1:** Hydrogen-bond geometry (Å, °)

*D*—H⋯*A*	*D*—H	H⋯*A*	*D*⋯*A*	*D*—H⋯*A*
O1*W*—H1*WA*⋯N12	0.82	2.43	3.106 (3)	140
O1*W*—H1*WB*⋯O4*W*	0.82	2.03	2.824 (4)	163
O2*W*—H2*WA*⋯O2^i^	0.82	1.96	2.746 (3)	160
O2*W*—H2*WB*⋯O7	0.82	2.24	3.051 (3)	169
O2*W*—H2*WB*⋯O8	0.82	2.58	3.091 (3)	122
N4—H4*A*⋯O8	0.87	2.05	2.805 (3)	144
O3*W*—H3*WA*⋯O5^i^	0.82	2.26	2.992 (4)	149
O3*W*—H3*WA*⋯O11^i^	0.82	2.44	3.008 (4)	127
O3*W*—H3*WB*⋯O10	0.82	2.12	2.890 (3)	155
O4*W*—H4*WA*⋯O4^ii^	0.82	2.06	2.858 (5)	166
N7—H7*A*⋯O2*W*	0.87	2.06	2.921 (3)	168
N7—H7*B*⋯O4^iii^	0.87	2.09	2.949 (3)	169
O4*W*—H4*WB*⋯O2^iv^	0.82	2.46	3.283 (4)	180
N8—H8*A*⋯O1*W*	0.87	2.10	2.813 (3)	139
N8—H8*B*⋯O8	0.87	1.94	2.762 (3)	156
N9—H9*A*⋯O2^v^	0.87	2.23	2.944 (3)	139
N9—H9*B*⋯N10^vi^	0.87	2.08	2.947 (3)	176
N11—H11*A*⋯O9	0.87	2.42	3.134 (3)	139
N13—H13*A*⋯O4^iv^	0.87	2.32	2.957 (3)	130
N13—H13*B*⋯N6^vi^	0.87	2.10	2.958 (3)	170
N14—H14*A*⋯O1^i^	0.87	2.54	3.398 (6)	169
N14—H14*A*⋯O2^i^	0.87	2.48	3.167 (6)	136
N14—H14*B*⋯O3*W*	0.87	2.55	3.119 (6)	124
N14—H14*B*⋯O9	0.87	2.51	3.121 (6)	127
N14—H14*B*⋯O10	0.87	2.42	3.228 (5)	156
N15—H15*A*⋯O6^vii^	0.87	1.94	2.797 (3)	166
N15—H15*B*⋯O12^iv^	0.87	2.06	2.911 (3)	167
C3—H3⋯O2*W*^viii^	0.95	2.36	3.130 (3)	138
C12—H12⋯O1*W*	0.95	2.58	3.326 (3)	136
C17—H17⋯N12^ix^	0.95	2.48	3.417 (4)	169
C19—H19⋯O3*W*^x^	0.95	2.31	3.215 (4)	158

Symmetry codes: (i) 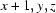; (ii) 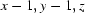; (iii) 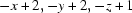; (iv) 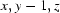; (v) 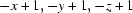; (vi) 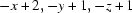; (vii) 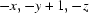; (viii) 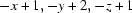; (ix) 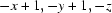; (x) 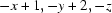.

**Fig. 1.**
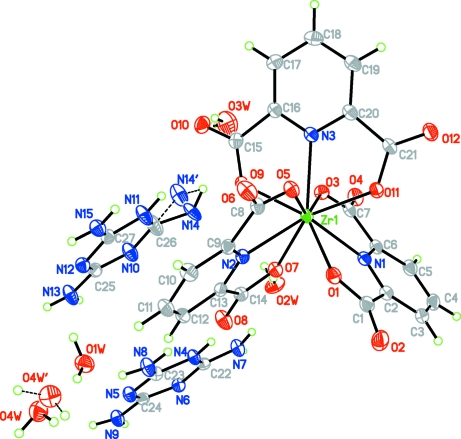
A view of the title compound showing thermal ellipsoids at the 50% probability level.

**Fig. 2.**
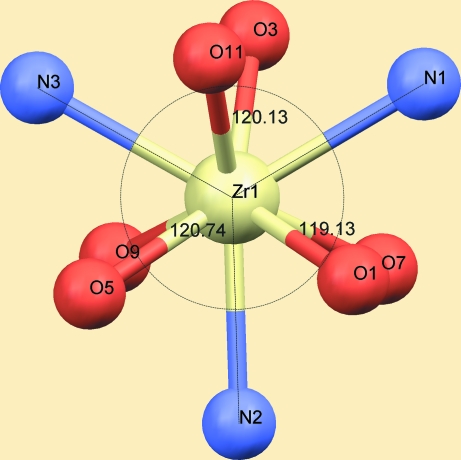
Coordination geometry (°) around the central atom.

**Fig. 3.**
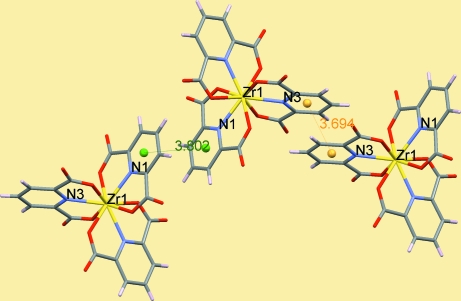
π-π stacking with centroid···centroid distances (Å).

**Fig. 4.**
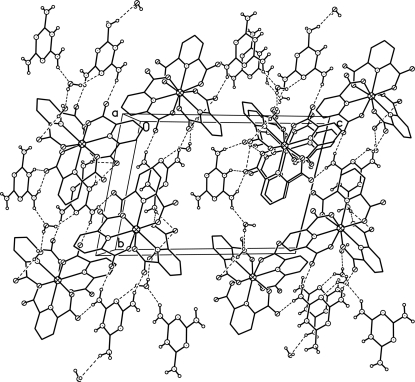
Crystal packing of the title compound along the crystallographic axis a. Hydrogen bonds are shown with dashed lines. Only the hydrogen atoms that take part in hydrogen bonding are depicted.

## supplementary crystallographic information

### Comment

Metal organic frameworks (MOFs) derived from proton transfer compounds are of interest in our team work. In this way, more than 160 compounds were synthesized and reported, most of which were reviewed in a recent paper (Aghabozorg *et al.*, 2008). Up to now, many nine-coordinated complexes of (pydc)^2-^ have been reported. For instance, a complex of Zr^IV^, [bis(oxyiminodiacetate)aquazirconate(IV)]^2-^, has been reported (Harben *et al.*, 2004). We have reported a nine-coordinated Zr^IV^, very similar to the title compound, formulated as (pydaH)_2_[Zr(pydc)_3_].5H_2_O (Aghabozorg *et al.*, 2005). Also, a nine-coordinated Y^III^ compound, (phenH)_3_[Y(pydc)_3_].DMSO.5H_2_O (phen is 1,10-phenanthroline) (Soleimannejad *et al.*, 2007), has been synthesized. The molecular structure of the title compound illustrated in Fig. 1 consists of three (pydc)^2– ^coordinated to Zr^IV^, two (tataH)^+ ^groups and four water molecules. The central atom is nine-coordinated by O1, O3, N1; O5, O7, N2 and O9, O11, N3 atoms of three (pydc)^2– ^groups. Studying the angles around Zr^IV ^reveals that sum of the angles between N1, N2 and N3 equals exactly to 360°. Therefore, Zr1 lies in the center of the N1/N2/N3 plane, and, as shown in Fig. 2, the coordination polyhedron is distorted tricapped trigonal prismatic. The metal–ligand bond distances are consistent with those found in (pydaH)_2_[Zr(pydc)_3_].5H_2_O (Aghabozorg *et al.*, 2005). The structure has numerous intermolecular interactions including C—O···π (with O···π distance of 3.893 (2) Å, π-π stacking (with centroid to centroid distances of 3.694 (2) and 3.802 (2) Å) (Fig. 3) as well as strong and weak hydrogen bonds (Table 1 and Fig. 4).

### Experimental

By refluxing 1 mmol (0.167 g) pyridine-2,6-dicarboxylic acid (pydcH_2_) and 1 mmol (0.126 g) 2,4,6-triamino-1,3,5-triazine (tata) in 150 ml water for 1.5 h, then adding 0.33 mmol (0.107 g) zirconyl chloride octahydrate (ZrOCl_2_.8H_2_O) and continuing to reflux for 1.5 h at 70°C, a cloudy solution was obtained. On refluxing the solution without heating for 3 h, it became completely clear and allowing it to concentrate at room temperature, colourless prismatic crystals were obtained after three weeks. The crystals were decomposed at 583 K.

### Refinement

The hydrogen atoms of NH groups and water molecules were found in difference Fourier synthesis. Except for two disordered groups, N—H and O—H distances were normalized to 0.87 and 0.82 Å, respectively, and the hydrogen atoms treated as riding on their bonded atoms. The H(C) atom positions were positioned geometrically with C—H = 0.95 Å. All hydrogen atoms were refined with isotropic thermal parameters having *U*_iso_(H) equal to 1.2 *U*_eq_ of the bonded atom. One of the NH_2_ groups showed large thermal motion and was split into two sites, N14 and N14', with occupancies fixed at 0.60 and 0.40, respectively. The two hydrogen atoms are shared by these two atoms and they were fixed at the positions that were found in a difference Fourier map. One of the water molecules is also disordered into two sites with occupancies of 0.7:0.3 selected such that almost equal Uiso's for O4w and O4w' were achieved. Four hydrogen atoms were located for these two O atoms but only those for the major site were normalized.

### Crystal data

**Table:**

(C_3_H_7_N_6_)_2_[Zr(C_7_H_3_NO_4_)_3_]·4H_2_O	*Z* = 2
*M**_r_* = 912.89	*F*(000) = 932
Triclinic, *P*1	*D*_x_ = 1.749 Mg m^−^^3^
Hall symbol: -P 1	Mo *K*α radiation, λ = 0.71073 Å
*a* = 9.3749 (16) Å	Cell parameters from 658 reflections
*b* = 12.308 (3) Å	θ = 3–28°
*c* = 16.934 (4) Å	µ = 0.42 mm^−^^1^
α = 97.926 (19)°	*T* = 120 K
β = 106.050 (12)°	Prism, colourless
γ = 107.839 (11)°	0.40 × 0.20 × 0.15 mm
*V* = 1733.8 (7) Å^3^	

### Data collection

**Table:**

Bruker SMART 1000 CCD area-detector diffractometer	8355 independent reflections
Radiation source: fine-focus sealed tube	6766 reflections with *I* > 2σ(*I*)
graphite	*R*_int_ = 0.027
φ and ω scans	θ_max_ = 28.0°, θ_min_ = 1.8°
Absorption correction: multi-scan (SADABS; Sheldrick, 1996)	*h* = −12→12
*T*_min_ = 0.851, *T*_max_ = 0.940	*k* = −16→16
17993 measured reflections	*l* = −22→22

### Refinement

**Table:**

Refinement on *F*^2^	Primary atom site location: structure-invariant direct methods
Least-squares matrix: full	Secondary atom site location: difference Fourier map
*R*[*F*^2^ > 2σ(*F*^2^)] = 0.041	Hydrogen site location: mixed
*wR*(*F*^2^) = 0.104	H-atom parameters constrained
*S* = 1.00	*w* = 1/[σ^2^(*F*_o_^2^) + (0.0505*P*)^2^ + 1.7*P*] where *P* = (*F*_o_^2^ + 2*F*_c_^2^)/3
8355 reflections	(Δ/σ)_max_ = 0.003
545 parameters	Δρ_max_ = 1.00 e Å^−^^3^
0 restraints	Δρ_min_ = −0.77 e Å^−^^3^

### Special details

**Table:**

Geometry. All e.s.d.'s (except the e.s.d. in the dihedral angle between two l.s. planes) are estimated using the full covariance matrix. The cell e.s.d.'s are taken into account individually in the estimation of e.s.d.'s in distances, angles and torsion angles; correlations between e.s.d.'s in cell parameters are only used when they are defined by crystal symmetry. An approximate (isotropic) treatment of cell e.s.d.'s is used for estimating e.s.d.'s involving l.s. planes.
Refinement. Refinement of *F*^2^ against ALL reflections. The weighted *R*-factor *wR* and goodness of fit *S* are based on *F*^2^, conventional *R*-factors *R* are based on *F*, with *F* set to zero for negative *F*^2^. The threshold expression of *F*^2^ > σ(*F*^2^) is used only for calculating *R*-factors(gt) *etc*. and is not relevant to the choice of reflections for refinement. *R*-factors based on *F*^2^ are statistically about twice as large as those based on *F*, and *R*- factors based on ALL data will be even larger.

### Fractional atomic coordinates and isotropic or equivalent isotropic displacement parameters (Å^2^)

**Table:**

	*x*	*y*	*z*	*U*_iso_*/*U*_eq_	Occ. (<1)
Zr1	0.28559 (3)	0.802835 (18)	0.195072 (14)	0.01451 (7)	
N1	0.3709 (2)	0.94504 (17)	0.32273 (12)	0.0168 (4)	
O1	0.10400 (19)	0.77557 (14)	0.25919 (11)	0.0198 (3)	
O2	0.0216 (2)	0.82091 (17)	0.36581 (14)	0.0340 (5)	
O3	0.53253 (19)	0.93108 (14)	0.22590 (10)	0.0185 (3)	
O4	0.7468 (2)	1.08521 (15)	0.30894 (11)	0.0231 (4)	
C1	0.1197 (3)	0.8424 (2)	0.32888 (16)	0.0212 (5)	
C2	0.2728 (3)	0.9455 (2)	0.36620 (15)	0.0187 (5)	
C3	0.3136 (3)	1.0328 (2)	0.43831 (16)	0.0234 (5)	
H3	0.2414	1.0317	0.4683	0.028*	
C4	0.4617 (3)	1.1214 (2)	0.46564 (16)	0.0236 (5)	
H4	0.4925	1.1828	0.5146	0.028*	
C5	0.5654 (3)	1.1200 (2)	0.42086 (15)	0.0221 (5)	
H5	0.6682	1.1796	0.4390	0.026*	
C6	0.5155 (3)	1.0298 (2)	0.34937 (15)	0.0173 (5)	
C7	0.6084 (3)	1.0150 (2)	0.29175 (15)	0.0175 (5)	
N2	0.1737 (2)	0.60123 (16)	0.19282 (12)	0.0155 (4)	
O5	0.05699 (19)	0.70749 (14)	0.08778 (10)	0.0182 (3)	
O6	−0.1565 (2)	0.54563 (15)	0.01431 (11)	0.0239 (4)	
O7	0.43417 (19)	0.74858 (14)	0.30198 (10)	0.0183 (3)	
O8	0.4862 (2)	0.61348 (15)	0.36795 (11)	0.0265 (4)	
C8	−0.0300 (3)	0.5985 (2)	0.07296 (15)	0.0171 (5)	
C9	0.0356 (3)	0.5343 (2)	0.13438 (14)	0.0165 (4)	
C10	−0.0384 (3)	0.4166 (2)	0.13214 (16)	0.0216 (5)	
H10	−0.1381	0.3702	0.0899	0.026*	
C11	0.0358 (3)	0.3686 (2)	0.19240 (16)	0.0221 (5)	
H11	−0.0119	0.2884	0.1923	0.027*	
C12	0.1816 (3)	0.4392 (2)	0.25330 (15)	0.0182 (5)	
H12	0.2354	0.4081	0.2954	0.022*	
C13	0.2465 (3)	0.5554 (2)	0.25141 (14)	0.0158 (4)	
C14	0.4018 (3)	0.6443 (2)	0.31261 (14)	0.0169 (5)	
N3	0.3092 (2)	0.86076 (16)	0.07171 (12)	0.0160 (4)	
O9	0.39755 (19)	0.70295 (14)	0.12953 (10)	0.0177 (3)	
O10	0.5097 (2)	0.67269 (16)	0.03268 (11)	0.0250 (4)	
O11	0.1938 (2)	0.94406 (14)	0.17254 (10)	0.0190 (3)	
O12	0.1385 (2)	1.07302 (15)	0.09910 (11)	0.0233 (4)	
C15	0.4337 (3)	0.7215 (2)	0.06333 (15)	0.0172 (5)	
C16	0.3755 (3)	0.80986 (19)	0.02482 (14)	0.0165 (4)	
C17	0.3836 (3)	0.8366 (2)	−0.05096 (15)	0.0202 (5)	
H17	0.4343	0.8016	−0.0827	0.024*	
C18	0.3155 (3)	0.9160 (2)	−0.07950 (16)	0.0225 (5)	
H18	0.3163	0.9341	−0.1322	0.027*	
C19	0.2463 (3)	0.9689 (2)	−0.03055 (15)	0.0206 (5)	
H19	0.1992	1.0233	−0.0490	0.025*	
C20	0.2480 (3)	0.9398 (2)	0.04572 (15)	0.0175 (5)	
C21	0.1862 (3)	0.9920 (2)	0.10879 (15)	0.0167 (5)	
N4	0.7448 (2)	0.58832 (17)	0.48483 (12)	0.0175 (4)	
H4A	0.6960	0.6270	0.4554	0.021*	
N5	0.7533 (2)	0.40707 (17)	0.51036 (13)	0.0194 (4)	
N6	0.9696 (2)	0.58949 (17)	0.58959 (12)	0.0184 (4)	
N7	0.9521 (2)	0.76211 (18)	0.55671 (14)	0.0227 (4)	
H7A	0.8982	0.7924	0.5217	0.027*	
H7B	1.0470	0.8008	0.5934	0.027*	
N8	0.5335 (2)	0.41661 (18)	0.41288 (13)	0.0234 (5)	
H8A	0.4855	0.3406	0.3967	0.028*	
H8B	0.4936	0.4635	0.3884	0.028*	
N9	0.9768 (2)	0.41118 (18)	0.61023 (13)	0.0224 (4)	
H9A	0.9358	0.3348	0.5938	0.027*	
H9B	1.0659	0.4460	0.6523	0.027*	
C22	0.8915 (3)	0.6470 (2)	0.54501 (14)	0.0170 (5)	
C23	0.6774 (3)	0.4676 (2)	0.46958 (15)	0.0187 (5)	
C24	0.8975 (3)	0.4710 (2)	0.56878 (14)	0.0181 (5)	
N10	0.7164 (3)	0.48215 (18)	0.24952 (14)	0.0253 (5)	
N11	0.4934 (3)	0.48189 (19)	0.14323 (14)	0.0271 (5)	
H11A	0.4506	0.5220	0.1122	0.032*	
N12	0.4830 (2)	0.30662 (17)	0.18547 (13)	0.0192 (4)	
N13	0.7066 (3)	0.30818 (19)	0.28279 (14)	0.0268 (5)	
H13A	0.6542	0.2336	0.2754	0.032*	
H13B	0.8004	0.3462	0.3207	0.032*	
N14	0.7048 (6)	0.6558 (5)	0.2174 (3)	0.0262 (11)	0.60
N14'	0.7338 (10)	0.6400 (7)	0.1863 (5)	0.0341 (19)	0.40
H14A	0.8089	0.6854	0.2358	0.041*	
H14B	0.6659	0.6837	0.1755	0.041*	
N15	0.2681 (2)	0.31340 (18)	0.08405 (13)	0.0207 (4)	
H15A	0.2180	0.3501	0.0532	0.025*	
H15B	0.2240	0.2385	0.0796	0.025*	
C25	0.6329 (3)	0.3668 (2)	0.23774 (15)	0.0188 (5)	
C26	0.6431 (3)	0.5381 (2)	0.20080 (19)	0.0322 (6)	
C27	0.4140 (3)	0.3657 (2)	0.13757 (15)	0.0180 (5)	
O1W	0.2873 (2)	0.21473 (16)	0.29928 (12)	0.0313 (4)	
H1WA	0.3023	0.2068	0.2537	0.038*	
H1WB	0.1976	0.1718	0.2947	0.038*	
O2W	0.7796 (2)	0.84349 (18)	0.42014 (12)	0.0296 (4)	
H2WA	0.8454	0.8469	0.3963	0.036*	
H2WB	0.6916	0.8170	0.3831	0.036*	
O3W	0.8371 (3)	0.8297 (2)	0.11479 (16)	0.0483 (6)	
H3WA	0.9164	0.8118	0.1270	0.058*	
H3WB	0.7562	0.7779	0.0809	0.058*	
O4W	−0.0041 (4)	0.0297 (3)	0.2674 (2)	0.0310 (7)	0.70
H4WA	−0.0839	0.0422	0.2701	0.037*	0.70
H4WB	0.0024	−0.0225	0.2920	0.037*	0.70
O4W'	−0.0135 (12)	0.0610 (8)	0.2484 (6)	0.036 (2)*	0.30
H4WC	−0.0592	−0.0132	0.2082	0.043*	0.30
H4WD	−0.0861	0.0687	0.2662	0.043*	0.30

### Atomic displacement parameters (Å^2^)

**Table:**

	*U*^11^	*U*^22^	*U*^33^	*U*^12^	*U*^13^	*U*^23^
Zr1	0.01427 (12)	0.01246 (11)	0.01488 (12)	0.00449 (8)	0.00258 (8)	0.00297 (8)
N1	0.0181 (10)	0.0154 (9)	0.0170 (9)	0.0068 (8)	0.0046 (8)	0.0053 (8)
O1	0.0185 (8)	0.0156 (8)	0.0245 (9)	0.0049 (7)	0.0072 (7)	0.0056 (7)
O2	0.0375 (11)	0.0250 (10)	0.0528 (13)	0.0126 (9)	0.0328 (10)	0.0119 (9)
O3	0.0166 (8)	0.0174 (8)	0.0190 (8)	0.0034 (7)	0.0055 (7)	0.0036 (7)
O4	0.0147 (8)	0.0183 (8)	0.0298 (10)	0.0013 (7)	0.0040 (7)	0.0038 (7)
C1	0.0221 (12)	0.0222 (12)	0.0276 (13)	0.0131 (10)	0.0130 (10)	0.0110 (10)
C2	0.0225 (12)	0.0170 (11)	0.0196 (11)	0.0090 (10)	0.0083 (10)	0.0067 (9)
C3	0.0348 (14)	0.0218 (12)	0.0222 (12)	0.0168 (11)	0.0135 (11)	0.0090 (10)
C4	0.0340 (14)	0.0179 (12)	0.0173 (12)	0.0126 (11)	0.0045 (10)	0.0013 (9)
C5	0.0252 (13)	0.0163 (11)	0.0202 (12)	0.0064 (10)	0.0030 (10)	0.0026 (9)
C6	0.0184 (11)	0.0145 (11)	0.0176 (11)	0.0066 (9)	0.0026 (9)	0.0049 (9)
C7	0.0150 (11)	0.0138 (11)	0.0223 (12)	0.0055 (9)	0.0028 (9)	0.0065 (9)
N2	0.0149 (9)	0.0148 (9)	0.0158 (9)	0.0054 (7)	0.0042 (8)	0.0030 (7)
O5	0.0159 (8)	0.0158 (8)	0.0197 (8)	0.0049 (6)	0.0016 (7)	0.0050 (7)
O6	0.0185 (9)	0.0206 (9)	0.0229 (9)	0.0028 (7)	−0.0035 (7)	0.0065 (7)
O7	0.0173 (8)	0.0168 (8)	0.0181 (8)	0.0053 (7)	0.0028 (7)	0.0040 (6)
O8	0.0228 (9)	0.0214 (9)	0.0255 (9)	0.0067 (7)	−0.0055 (7)	0.0069 (7)
C8	0.0162 (11)	0.0155 (11)	0.0184 (11)	0.0064 (9)	0.0039 (9)	0.0031 (9)
C9	0.0175 (11)	0.0169 (11)	0.0160 (11)	0.0082 (9)	0.0047 (9)	0.0037 (9)
C10	0.0167 (11)	0.0186 (12)	0.0215 (12)	0.0010 (9)	0.0008 (10)	0.0034 (10)
C11	0.0224 (12)	0.0155 (11)	0.0259 (13)	0.0038 (10)	0.0068 (10)	0.0069 (10)
C12	0.0202 (11)	0.0186 (11)	0.0177 (11)	0.0091 (9)	0.0059 (9)	0.0065 (9)
C13	0.0178 (11)	0.0152 (11)	0.0152 (11)	0.0073 (9)	0.0057 (9)	0.0032 (9)
C14	0.0155 (11)	0.0184 (11)	0.0166 (11)	0.0066 (9)	0.0045 (9)	0.0043 (9)
N3	0.0137 (9)	0.0131 (9)	0.0166 (9)	0.0022 (7)	0.0013 (8)	0.0031 (7)
O9	0.0191 (8)	0.0158 (8)	0.0182 (8)	0.0076 (7)	0.0047 (7)	0.0047 (6)
O10	0.0261 (9)	0.0282 (10)	0.0257 (9)	0.0157 (8)	0.0102 (8)	0.0055 (8)
O11	0.0226 (9)	0.0161 (8)	0.0188 (8)	0.0083 (7)	0.0061 (7)	0.0045 (7)
O12	0.0268 (9)	0.0198 (9)	0.0255 (9)	0.0118 (7)	0.0074 (8)	0.0075 (7)
C15	0.0129 (10)	0.0157 (11)	0.0164 (11)	0.0022 (9)	0.0004 (9)	−0.0003 (9)
C16	0.0137 (10)	0.0133 (10)	0.0171 (11)	0.0017 (8)	0.0018 (9)	0.0017 (9)
C17	0.0182 (11)	0.0208 (12)	0.0187 (11)	0.0039 (9)	0.0064 (9)	0.0025 (9)
C18	0.0205 (12)	0.0242 (12)	0.0181 (12)	0.0032 (10)	0.0039 (10)	0.0075 (10)
C19	0.0182 (11)	0.0184 (11)	0.0215 (12)	0.0042 (9)	0.0023 (10)	0.0073 (10)
C20	0.0152 (11)	0.0147 (11)	0.0187 (11)	0.0031 (9)	0.0022 (9)	0.0043 (9)
C21	0.0141 (10)	0.0132 (10)	0.0200 (11)	0.0039 (9)	0.0021 (9)	0.0059 (9)
N4	0.0147 (9)	0.0154 (9)	0.0205 (10)	0.0067 (8)	0.0010 (8)	0.0056 (8)
N5	0.0177 (10)	0.0166 (10)	0.0215 (10)	0.0062 (8)	0.0030 (8)	0.0047 (8)
N6	0.0176 (10)	0.0188 (10)	0.0163 (9)	0.0065 (8)	0.0024 (8)	0.0035 (8)
N7	0.0186 (10)	0.0169 (10)	0.0255 (11)	0.0052 (8)	−0.0015 (9)	0.0041 (8)
N8	0.0189 (10)	0.0164 (10)	0.0274 (11)	0.0048 (8)	−0.0009 (9)	0.0040 (9)
N9	0.0209 (10)	0.0189 (10)	0.0234 (11)	0.0086 (8)	0.0000 (9)	0.0054 (8)
C22	0.0150 (11)	0.0202 (11)	0.0154 (11)	0.0057 (9)	0.0053 (9)	0.0043 (9)
C23	0.0178 (11)	0.0183 (11)	0.0195 (11)	0.0064 (9)	0.0062 (9)	0.0041 (9)
C24	0.0201 (11)	0.0217 (12)	0.0154 (11)	0.0100 (10)	0.0069 (9)	0.0058 (9)
N10	0.0204 (11)	0.0191 (10)	0.0304 (12)	0.0064 (9)	−0.0007 (9)	0.0088 (9)
N11	0.0239 (11)	0.0189 (10)	0.0312 (12)	0.0078 (9)	−0.0038 (9)	0.0106 (9)
N12	0.0190 (10)	0.0186 (10)	0.0183 (10)	0.0074 (8)	0.0030 (8)	0.0048 (8)
N13	0.0228 (11)	0.0167 (10)	0.0307 (12)	0.0057 (9)	−0.0053 (9)	0.0071 (9)
N14	0.023 (2)	0.018 (2)	0.027 (3)	0.0026 (17)	−0.0054 (19)	0.012 (2)
N14'	0.026 (4)	0.023 (3)	0.042 (5)	0.006 (3)	−0.003 (4)	0.009 (4)
N15	0.0185 (10)	0.0209 (10)	0.0211 (10)	0.0080 (8)	0.0023 (8)	0.0070 (8)
C25	0.0201 (12)	0.0199 (12)	0.0175 (11)	0.0094 (9)	0.0056 (9)	0.0051 (9)
C26	0.0252 (14)	0.0218 (13)	0.0381 (16)	0.0055 (11)	−0.0053 (12)	0.0112 (12)
C27	0.0184 (11)	0.0185 (11)	0.0174 (11)	0.0080 (9)	0.0056 (9)	0.0034 (9)
O1W	0.0360 (11)	0.0244 (10)	0.0247 (10)	0.0027 (8)	0.0064 (8)	0.0052 (8)
O2W	0.0213 (9)	0.0449 (12)	0.0224 (9)	0.0128 (9)	0.0080 (8)	0.0040 (8)
O3W	0.0403 (13)	0.0441 (13)	0.0590 (16)	0.0177 (11)	0.0113 (12)	0.0140 (12)
O4W	0.0331 (17)	0.0289 (17)	0.0353 (18)	0.0144 (14)	0.0147 (14)	0.0079 (15)

### Geometric parameters (Å, °)

**Table:**

Zr1—O11	2.1964 (16)	C19—C20	1.384 (3)
Zr1—O9	2.2144 (17)	C19—H19	0.9500
Zr1—O1	2.2276 (17)	C20—C21	1.507 (3)
Zr1—O3	2.2337 (17)	N4—C22	1.366 (3)
Zr1—O5	2.2336 (17)	N4—C23	1.380 (3)
Zr1—O7	2.2743 (17)	N4—H4A	0.8700
Zr1—N3	2.343 (2)	N5—C23	1.316 (3)
Zr1—N1	2.349 (2)	N5—C24	1.349 (3)
Zr1—N2	2.370 (2)	N6—C22	1.329 (3)
N1—C2	1.329 (3)	N6—C24	1.353 (3)
N1—C6	1.340 (3)	N7—C22	1.318 (3)
O1—C1	1.286 (3)	N7—H7A	0.8700
O2—C1	1.233 (3)	N7—H7B	0.8700
O3—C7	1.269 (3)	N8—C23	1.318 (3)
O4—C7	1.244 (3)	N8—H8A	0.8700
C1—C2	1.494 (3)	N8—H8B	0.8701
C2—C3	1.386 (3)	N9—C24	1.333 (3)
C3—C4	1.382 (4)	N9—H9A	0.8700
C3—H3	0.9500	N9—H9B	0.8700
C4—C5	1.391 (4)	N10—C26	1.327 (3)
C4—H4	0.9500	N10—C25	1.351 (3)
C5—C6	1.383 (3)	N11—C26	1.364 (3)
C5—H5	0.9500	N11—C27	1.371 (3)
C6—C7	1.504 (3)	N11—H11A	0.8700
N2—C9	1.322 (3)	N12—C27	1.329 (3)
N2—C13	1.335 (3)	N12—C25	1.342 (3)
O5—C8	1.285 (3)	N13—C25	1.321 (3)
O6—C8	1.230 (3)	N13—H13A	0.8699
O7—C14	1.275 (3)	N13—H13B	0.8700
O8—C14	1.233 (3)	N14—C26	1.340 (6)
C8—C9	1.502 (3)	N14—H14A	0.8749
C9—C10	1.392 (3)	N14—H14B	0.8687
C10—C11	1.379 (3)	N14'—C26	1.380 (9)
C10—H10	0.9500	N14'—H14A	0.9028
C11—C12	1.390 (3)	N14'—H14B	0.9488
C11—H11	0.9500	N15—C27	1.312 (3)
C12—C13	1.382 (3)	N15—H15A	0.8700
C12—H12	0.9500	N15—H15B	0.8700
C13—C14	1.506 (3)	O1W—H1WA	0.8200
N3—C16	1.335 (3)	O1W—H1WB	0.8201
N3—C20	1.338 (3)	O2W—H2WA	0.8199
O9—C15	1.287 (3)	O2W—H2WB	0.8200
O10—C15	1.233 (3)	O3W—H3WA	0.8199
O11—C21	1.295 (3)	O3W—H3WB	0.8200
O12—C21	1.224 (3)	O4W—H4WA	0.8199
C15—C16	1.506 (3)	O4W—H4WB	0.8201
C16—C17	1.384 (3)	O4W—H4WC	0.9759
C17—C18	1.392 (4)	O4W—H4WD	1.0210
C17—H17	0.9500	O4W'—H4WA	0.8285
C18—C19	1.393 (4)	O4W'—H4WC	0.9537
C18—H18	0.9500	O4W'—H4WD	0.8410
			
O11—Zr1—O9	135.20 (6)	O8—C14—C13	119.7 (2)
O11—Zr1—O1	77.18 (6)	O7—C14—C13	115.10 (19)
O9—Zr1—O1	140.70 (6)	C16—N3—C20	120.0 (2)
O11—Zr1—O3	89.27 (6)	C16—N3—Zr1	119.94 (15)
O9—Zr1—O3	76.40 (6)	C20—N3—Zr1	119.92 (16)
O1—Zr1—O3	134.77 (6)	C15—O9—Zr1	125.29 (14)
O11—Zr1—O5	77.71 (6)	C21—O11—Zr1	127.15 (15)
O9—Zr1—O5	88.12 (6)	O10—C15—O9	124.2 (2)
O1—Zr1—O5	77.03 (6)	O10—C15—C16	121.4 (2)
O3—Zr1—O5	142.19 (6)	O9—C15—C16	114.4 (2)
O11—Zr1—O7	140.58 (6)	N3—C16—C17	121.8 (2)
O9—Zr1—O7	77.15 (6)	N3—C16—C15	112.2 (2)
O1—Zr1—O7	87.39 (6)	C17—C16—C15	126.0 (2)
O3—Zr1—O7	76.22 (6)	C16—C17—C18	118.3 (2)
O5—Zr1—O7	134.02 (6)	C16—C17—H17	120.9
O11—Zr1—N3	67.37 (7)	C18—C17—H17	120.9
O9—Zr1—N3	67.83 (6)	C17—C18—C19	119.8 (2)
O1—Zr1—N3	135.82 (6)	C17—C18—H18	120.1
O3—Zr1—N3	71.70 (6)	C19—C18—H18	120.1
O5—Zr1—N3	70.52 (6)	C20—C19—C18	118.0 (2)
O7—Zr1—N3	136.80 (6)	C20—C19—H19	121.0
O11—Zr1—N1	70.33 (6)	C18—C19—H19	121.0
O9—Zr1—N1	135.65 (6)	N3—C20—C19	122.0 (2)
O1—Zr1—N1	67.44 (7)	N3—C20—C21	112.4 (2)
O3—Zr1—N1	67.36 (7)	C19—C20—C21	125.6 (2)
O5—Zr1—N1	136.22 (7)	O12—C21—O11	125.7 (2)
O7—Zr1—N1	70.25 (6)	O12—C21—C20	121.5 (2)
N3—Zr1—N1	120.12 (7)	O11—C21—C20	112.80 (19)
O11—Zr1—N2	135.82 (7)	C22—N4—C23	119.5 (2)
O9—Zr1—N2	71.12 (6)	C22—N4—H4A	119.9
O1—Zr1—N2	69.59 (6)	C23—N4—H4A	120.6
O3—Zr1—N2	134.88 (6)	C23—N5—C24	115.7 (2)
O5—Zr1—N2	67.27 (6)	C22—N6—C24	115.8 (2)
O7—Zr1—N2	66.76 (6)	C22—N7—H7A	116.1
N3—Zr1—N2	120.74 (7)	C22—N7—H7B	118.8
N1—Zr1—N2	119.13 (7)	H7A—N7—H7B	124.5
C2—N1—C6	119.8 (2)	C23—N8—H8A	122.4
C2—N1—Zr1	120.07 (16)	C23—N8—H8B	115.6
C6—N1—Zr1	120.01 (15)	H8A—N8—H8B	121.7
C1—O1—Zr1	124.99 (15)	C24—N9—H9A	117.4
C7—O3—Zr1	125.60 (15)	C24—N9—H9B	122.3
O2—C1—O1	123.5 (2)	H9A—N9—H9B	120.3
O2—C1—C2	121.4 (2)	N7—C22—N6	121.6 (2)
O1—C1—C2	114.9 (2)	N7—C22—N4	117.4 (2)
N1—C2—C3	122.1 (2)	N6—C22—N4	120.9 (2)
N1—C2—C1	112.4 (2)	N5—C23—N8	122.0 (2)
C3—C2—C1	125.6 (2)	N5—C23—N4	121.4 (2)
C4—C3—C2	118.5 (2)	N8—C23—N4	116.6 (2)
C4—C3—H3	120.8	N9—C24—N5	116.6 (2)
C2—C3—H3	120.8	N9—C24—N6	116.8 (2)
C3—C4—C5	119.4 (2)	N5—C24—N6	126.6 (2)
C3—C4—H4	120.3	C26—N10—C25	115.4 (2)
C5—C4—H4	120.3	C26—N11—C27	119.3 (2)
C6—C5—C4	118.5 (2)	C26—N11—H11A	118.4
C6—C5—H5	120.7	C27—N11—H11A	122.2
C4—C5—H5	120.7	C27—N12—C25	116.2 (2)
N1—C6—C5	121.7 (2)	C25—N13—H13A	117.1
N1—C6—C7	112.0 (2)	C25—N13—H13B	119.3
C5—C6—C7	126.3 (2)	H13A—N13—H13B	123.4
O4—C7—O3	125.2 (2)	C26—N14—H14A	114.1
O4—C7—C6	119.9 (2)	C26—N14—H14B	113.2
O3—C7—C6	114.8 (2)	H14A—N14—H14B	110.9
C9—N2—C13	119.7 (2)	C26—N14'—H14A	108.8
C9—N2—Zr1	119.59 (15)	C26—N14'—H14B	104.7
C13—N2—Zr1	120.67 (15)	H14A—N14'—H14B	101.7
C8—O5—Zr1	125.56 (14)	C27—N15—H15A	122.7
C14—O7—Zr1	125.03 (14)	C27—N15—H15B	116.3
O6—C8—O5	125.9 (2)	H15A—N15—H15B	121.0
O6—C8—C9	119.7 (2)	N13—C25—N12	117.2 (2)
O5—C8—C9	114.3 (2)	N13—C25—N10	116.3 (2)
N2—C9—C10	122.0 (2)	N12—C25—N10	126.5 (2)
N2—C9—C8	113.2 (2)	N10—C26—N14	119.5 (3)
C10—C9—C8	124.9 (2)	N10—C26—N11	121.7 (2)
C11—C10—C9	118.7 (2)	N14—C26—N11	117.9 (3)
C11—C10—H10	120.6	N10—C26—N14'	118.7 (4)
C9—C10—H10	120.6	N11—C26—N14'	115.9 (4)
C10—C11—C12	119.0 (2)	N15—C27—N12	120.3 (2)
C10—C11—H11	120.5	N15—C27—N11	118.9 (2)
C12—C11—H11	120.5	N12—C27—N11	120.8 (2)
C13—C12—C11	118.6 (2)	H1WA—O1W—H1WB	111.2
C13—C12—H12	120.7	H2WA—O2W—H2WB	107.1
C11—C12—H12	120.7	H3WA—O3W—H3WB	115.2
N2—C13—C12	121.9 (2)	H4WA—O4W—H4WB	106.9
N2—C13—C14	112.32 (19)	H4WB—O4W—H4WC	103.3
C12—C13—C14	125.7 (2)	H4WB—O4W—H4WD	123.7
O8—C14—O7	125.2 (2)	H4WC—O4W'—H4WD	106.2
			
O11—Zr1—N1—C2	79.81 (17)	N2—C9—C10—C11	−0.5 (4)
O9—Zr1—N1—C2	−144.77 (16)	C8—C9—C10—C11	179.7 (2)
O1—Zr1—N1—C2	−4.07 (16)	C9—C10—C11—C12	0.1 (4)
O3—Zr1—N1—C2	177.53 (19)	C10—C11—C12—C13	0.3 (4)
O5—Zr1—N1—C2	34.2 (2)	C9—N2—C13—C12	−0.1 (3)
O7—Zr1—N1—C2	−99.62 (18)	Zr1—N2—C13—C12	177.89 (17)
N3—Zr1—N1—C2	126.98 (17)	C9—N2—C13—C14	−179.9 (2)
N2—Zr1—N1—C2	−52.46 (19)	Zr1—N2—C13—C14	−1.9 (3)
O11—Zr1—N1—C6	−96.45 (17)	C11—C12—C13—N2	−0.3 (4)
O9—Zr1—N1—C6	39.0 (2)	C11—C12—C13—C14	179.4 (2)
O1—Zr1—N1—C6	179.67 (18)	Zr1—O7—C14—O8	176.13 (18)
O3—Zr1—N1—C6	1.27 (15)	Zr1—O7—C14—C13	−4.2 (3)
O5—Zr1—N1—C6	−142.04 (15)	N2—C13—C14—O8	−176.6 (2)
O7—Zr1—N1—C6	84.13 (17)	C12—C13—C14—O8	3.7 (4)
N3—Zr1—N1—C6	−49.28 (18)	N2—C13—C14—O7	3.7 (3)
N2—Zr1—N1—C6	131.28 (16)	C12—C13—C14—O7	−176.0 (2)
O11—Zr1—O1—C1	−72.21 (18)	O11—Zr1—N3—C16	178.94 (18)
O9—Zr1—O1—C1	137.22 (17)	O9—Zr1—N3—C16	−0.52 (15)
O3—Zr1—O1—C1	3.7 (2)	O1—Zr1—N3—C16	−141.75 (15)
O5—Zr1—O1—C1	−152.33 (19)	O3—Zr1—N3—C16	81.86 (17)
O7—Zr1—O1—C1	71.25 (18)	O5—Zr1—N3—C16	−96.63 (17)
N3—Zr1—O1—C1	−109.06 (19)	O7—Zr1—N3—C16	37.8 (2)
N1—Zr1—O1—C1	1.57 (17)	N1—Zr1—N3—C16	130.51 (16)
N2—Zr1—O1—C1	137.39 (19)	N2—Zr1—N3—C16	−50.06 (18)
O11—Zr1—O3—C7	70.44 (18)	O11—Zr1—N3—C20	−4.98 (16)
O9—Zr1—O3—C7	−152.41 (18)	O9—Zr1—N3—C20	175.56 (18)
O1—Zr1—O3—C7	−0.6 (2)	O1—Zr1—N3—C20	34.3 (2)
O5—Zr1—O3—C7	139.11 (17)	O3—Zr1—N3—C20	−102.06 (17)
O7—Zr1—O3—C7	−72.56 (18)	O5—Zr1—N3—C20	79.44 (17)
N3—Zr1—O3—C7	136.80 (19)	O7—Zr1—N3—C20	−146.12 (15)
N1—Zr1—O3—C7	1.50 (17)	N1—Zr1—N3—C20	−53.42 (18)
N2—Zr1—O3—C7	−107.71 (18)	N2—Zr1—N3—C20	126.02 (16)
Zr1—O1—C1—O2	−175.06 (18)	O11—Zr1—O9—C15	3.7 (2)
Zr1—O1—C1—C2	0.7 (3)	O1—Zr1—O9—C15	140.88 (16)
C6—N1—C2—C3	1.3 (3)	O3—Zr1—O9—C15	−71.07 (17)
Zr1—N1—C2—C3	−175.02 (17)	O5—Zr1—O9—C15	74.13 (17)
C6—N1—C2—C1	−178.1 (2)	O7—Zr1—O9—C15	−149.76 (18)
Zr1—N1—C2—C1	5.7 (3)	N3—Zr1—O9—C15	4.43 (16)
O2—C1—C2—N1	171.7 (2)	N1—Zr1—O9—C15	−106.57 (18)
O1—C1—C2—N1	−4.2 (3)	N2—Zr1—O9—C15	140.72 (18)
O2—C1—C2—C3	−7.5 (4)	O9—Zr1—O11—C21	2.9 (2)
O1—C1—C2—C3	176.6 (2)	O1—Zr1—O11—C21	−150.93 (19)
N1—C2—C3—C4	−0.4 (4)	O3—Zr1—O11—C21	72.58 (18)
C1—C2—C3—C4	178.8 (2)	O5—Zr1—O11—C21	−71.65 (18)
C2—C3—C4—C5	−0.6 (4)	O7—Zr1—O11—C21	139.58 (17)
C3—C4—C5—C6	0.7 (4)	N3—Zr1—O11—C21	2.15 (17)
C2—N1—C6—C5	−1.1 (3)	N1—Zr1—O11—C21	138.73 (19)
Zr1—N1—C6—C5	175.18 (17)	N2—Zr1—O11—C21	−109.31 (19)
C2—N1—C6—C7	−179.5 (2)	Zr1—O9—C15—O10	172.67 (17)
Zr1—N1—C6—C7	−3.3 (2)	Zr1—O9—C15—C16	−7.2 (3)
C4—C5—C6—N1	0.1 (4)	C20—N3—C16—C17	0.2 (3)
C4—C5—C6—C7	178.3 (2)	Zr1—N3—C16—C17	176.24 (17)
Zr1—O3—C7—O4	179.05 (17)	C20—N3—C16—C15	−178.54 (19)
Zr1—O3—C7—C6	−3.7 (3)	Zr1—N3—C16—C15	−2.5 (2)
N1—C6—C7—O4	−178.3 (2)	O10—C15—C16—N3	−174.0 (2)
C5—C6—C7—O4	3.4 (4)	O9—C15—C16—N3	5.8 (3)
N1—C6—C7—O3	4.3 (3)	O10—C15—C16—C17	7.3 (4)
C5—C6—C7—O3	−174.1 (2)	O9—C15—C16—C17	−172.8 (2)
O11—Zr1—N2—C9	38.2 (2)	N3—C16—C17—C18	−2.1 (3)
O9—Zr1—N2—C9	−98.20 (17)	C15—C16—C17—C18	176.4 (2)
O1—Zr1—N2—C9	81.91 (17)	C16—C17—C18—C19	2.0 (4)
O3—Zr1—N2—C9	−144.46 (16)	C17—C18—C19—C20	0.0 (4)
O5—Zr1—N2—C9	−2.13 (16)	C16—N3—C20—C19	2.0 (3)
O7—Zr1—N2—C9	178.05 (18)	Zr1—N3—C20—C19	−174.09 (17)
N3—Zr1—N2—C9	−50.07 (19)	C16—N3—C20—C21	−177.09 (19)
N1—Zr1—N2—C9	129.38 (17)	Zr1—N3—C20—C21	6.8 (2)
O11—Zr1—N2—C13	−139.83 (16)	C18—C19—C20—N3	−2.0 (3)
O9—Zr1—N2—C13	83.78 (17)	C18—C19—C20—C21	176.9 (2)
O1—Zr1—N2—C13	−96.11 (18)	Zr1—O11—C21—O12	−177.80 (17)
O3—Zr1—N2—C13	37.5 (2)	Zr1—O11—C21—C20	0.5 (3)
O5—Zr1—N2—C13	179.84 (19)	N3—C20—C21—O12	173.6 (2)
O7—Zr1—N2—C13	0.03 (16)	C19—C20—C21—O12	−5.4 (4)
N3—Zr1—N2—C13	131.91 (16)	N3—C20—C21—O11	−4.8 (3)
N1—Zr1—N2—C13	−48.65 (19)	C19—C20—C21—O11	176.2 (2)
O11—Zr1—O5—C8	−150.83 (19)	C24—N6—C22—N7	−177.1 (2)
O9—Zr1—O5—C8	71.97 (18)	C24—N6—C22—N4	2.7 (3)
O1—Zr1—O5—C8	−71.37 (18)	C23—N4—C22—N7	179.2 (2)
O3—Zr1—O5—C8	136.75 (17)	C23—N4—C22—N6	−0.7 (3)
O7—Zr1—O5—C8	1.9 (2)	C24—N5—C23—N8	−177.7 (2)
N3—Zr1—O5—C8	139.08 (19)	C24—N5—C23—N4	1.7 (3)
N1—Zr1—O5—C8	−107.32 (19)	C22—N4—C23—N5	−1.7 (3)
N2—Zr1—O5—C8	1.68 (17)	C22—N4—C23—N8	177.7 (2)
O11—Zr1—O7—C14	137.40 (17)	C23—N5—C24—N9	−179.4 (2)
O9—Zr1—O7—C14	−72.30 (18)	C23—N5—C24—N6	0.7 (4)
O1—Zr1—O7—C14	71.31 (18)	C22—N6—C24—N9	177.2 (2)
O3—Zr1—O7—C14	−151.21 (19)	C22—N6—C24—N5	−2.8 (4)
O5—Zr1—O7—C14	2.2 (2)	C27—N12—C25—N13	−176.6 (2)
N3—Zr1—O7—C14	−108.38 (19)	C27—N12—C25—N10	3.8 (4)
N1—Zr1—O7—C14	138.25 (19)	C26—N10—C25—N13	176.9 (3)
N2—Zr1—O7—C14	2.44 (17)	C26—N10—C25—N12	−3.5 (4)
Zr1—O5—C8—O6	179.53 (18)	C25—N10—C26—N14	169.0 (3)
Zr1—O5—C8—C9	−1.1 (3)	C25—N10—C26—N11	0.2 (4)
C13—N2—C9—C10	0.6 (3)	C25—N10—C26—N14'	−157.4 (4)
Zr1—N2—C9—C10	−177.49 (18)	C27—N11—C26—N10	2.4 (4)
C13—N2—C9—C8	−179.6 (2)	C27—N11—C26—N14	−166.5 (3)
Zr1—N2—C9—C8	2.3 (3)	C27—N11—C26—N14'	160.7 (4)
O6—C8—C9—N2	178.5 (2)	C25—N12—C27—N15	179.4 (2)
O5—C8—C9—N2	−0.9 (3)	C25—N12—C27—N11	−0.8 (3)
O6—C8—C9—C10	−1.7 (4)	C26—N11—C27—N15	177.7 (3)
O5—C8—C9—C10	178.9 (2)	C26—N11—C27—N12	−2.1 (4)

### Hydrogen-bond geometry (Å, °)

**Table:**

*D*—H···*A*	*D*—H	H···*A*	*D*···*A*	*D*—H···*A*
O1W—H1WA···N12	0.82	2.43	3.106 (3)	140
O1W—H1WB···O4W	0.82	2.03	2.824 (4)	163
O2W—H2WA···O2^i^	0.82	1.96	2.746 (3)	160
O2W—H2WB···O7	0.82	2.24	3.051 (3)	169
O2W—H2WB···O8	0.82	2.58	3.091 (3)	122
N4—H4A···O8	0.87	2.05	2.805 (3)	144
O3W—H3WA···O5^i^	0.82	2.26	2.992 (4)	149
O3W—H3WA···O11^i^	0.82	2.44	3.008 (4)	127
O3W—H3WB···O10	0.82	2.12	2.890 (3)	155
O4W—H4WA···O4^ii^	0.82	2.06	2.858 (5)	166
N7—H7A···O2W	0.87	2.06	2.921 (3)	168
N7—H7B···O4^iii^	0.87	2.09	2.949 (3)	169
O4W—H4WB···O2^iv^	0.82	2.46	3.283 (4)	180
N8—H8A···O1W	0.87	2.10	2.813 (3)	139
N8—H8B···O8	0.87	1.94	2.762 (3)	156
N9—H9A···O2^v^	0.87	2.23	2.944 (3)	139
N9—H9B···N10^vi^	0.87	2.08	2.947 (3)	176
N11—H11A···O9	0.87	2.42	3.134 (3)	139
N13—H13A···O4^iv^	0.87	2.32	2.957 (3)	130
N13—H13B···N6^vi^	0.87	2.10	2.958 (3)	170
N14—H14A···O1^i^	0.87	2.54	3.398 (6)	169
N14—H14A···O2^i^	0.87	2.48	3.167 (6)	136
N14—H14B···O3W	0.87	2.55	3.119 (6)	124
N14—H14B···O9	0.87	2.51	3.121 (6)	127
N14—H14B···O10	0.87	2.42	3.228 (5)	156
N15—H15A···O6^vii^	0.87	1.94	2.797 (3)	166
N15—H15B···O12^iv^	0.87	2.06	2.911 (3)	167
C3—H3···O2W^viii^	0.95	2.36	3.130 (3)	138
C12—H12···O1W	0.95	2.58	3.326 (3)	136
C17—H17···N12^ix^	0.95	2.48	3.417 (4)	169
C19—H19···O3W^x^	0.95	2.31	3.215 (4)	158
C14—O8···Cg1	1.23	3.89	4.068 (3)	89

Symmetry codes:  (i) *x*+1, *y*, *z*; (ii) *x*−1, *y*−1, *z*; (iii) −*x*+2, −*y*+2, −*z*+1; (iv) *x*, *y*−1, *z*; (v) −*x*+1, −*y*+1, −*z*+1; (vi) −*x*+2, −*y*+1, −*z*+1; (vii) −*x*, −*y*+1, −*z*; (viii) −*x*+1, −*y*+2, −*z*+1; (ix) −*x*+1, −*y*+1, −*z*; (x) −*x*+1, −*y*+2, −*z*.
